# Purinergic signaling: Diverse effects and therapeutic potential in cancer

**DOI:** 10.3389/fonc.2023.1058371

**Published:** 2023-01-18

**Authors:** Jasmeet Kaur, Sanchit Dora

**Affiliations:** Department of Biophysics, Postgraduate Institute of Medical Education and Research (PGIMER), Chandigarh, India

**Keywords:** purinergic signaling, ATP, ADO, P1, P2X, P2Y, cancer

## Abstract

Regardless of improved biological insights and therapeutic advances, cancer is consuming multiple lives worldwide. Cancer is a complex disease with diverse cellular, metabolic, and physiological parameters as its hallmarks. This instigates a need to uncover the latest therapeutic targets to advance the treatment of cancer patients. Purines are building blocks of nucleic acids but also function as metabolic intermediates and messengers, as part of a signaling pathway known as purinergic signaling. Purinergic signaling comprises primarily adenosine triphosphate (ATP) and adenosine (ADO), their analogous membrane receptors, and a set of ectonucleotidases, and has both short- and long-term (trophic) effects. Cells release ATP and ADO to modulate cellular function in an autocrine or paracrine manner by activating membrane-localized purinergic receptors (purinoceptors, P1 and P2). P1 receptors are selective for ADO and have four recognized subtypes—A1, A2A, A2B, and A3. Purines and pyrimidines activate P2 receptors, and the P2X subtype is ligand-gated ion channel receptors. P2X has seven subtypes (P2X1–7) and forms homo- and heterotrimers. The P2Y subtype is a G protein-coupled receptor with eight subtypes (P2Y1/2/4/6/11/12/13/14). ATP, its derivatives, and purinoceptors are widely distributed in all cell types for cellular communication, and any imbalance compromises the homeostasis of the cell. Neurotransmission, neuromodulation, and secretion employ fast purinergic signaling, while trophic purinergic signaling regulates cell metabolism, proliferation, differentiation, survival, migration, invasion, and immune response during tumor progression. Thus, purinergic signaling is a prospective therapeutic target in cancer and therapy resistance.

## Introduction

Diverse cellular, molecular, and metabolic adequacies underlie cancer’s pathology (1–3). Aerobic glycolysis or the Warburg effect is a prominent metabolic feature in tumors (4, 5). Cancer cells acquire ATP from glycolysis preferably, in spite of ample oxygen availability and mitochondrial oxidative phosphorylation fitness (6).

Cancer cells are committed to replication and constantly use metabolic pathways to provide biomolecules and genetic material to promote their undifferentiated and aggressive state (7). Cancer cells constantly experience a “metabolic dilemma”: deciding whether to use ATP to facilitate biosynthetic processes/reductive power (NADPH) and biomolecules to synthesize membrane and genetic material (8). Thus, understanding the multifaceted metabolic adaptations in cancer is crucial to develop successful therapeutic approaches.

Purine nucleotides and nucleosides such as adenosine and ATP regulate intracellular energy homeostasis, nucleotide synthesis, and immune response (9, 10). Purines bind and activate plasmalemmal purinoceptors as endogenous ligands in the cell and/or in nearby cells and regulate extracellular signaling known as “purinergic signaling” (11). The Hungarian physiologist Albert Szent-Györgyi first described purines’ extracellular role, after he observed a temporary reduction in heart rate in a guinea pig by purified adenine compounds (12). In 1972, Geoffrey Burnstock proposed ATP as a non-adrenergic, non-cholinergic (NANC) neurotransmitter (11), but it seemed implausible that cells will actively release ATP as a neurotransmitter if it is already a ubiquitous intracellular molecular energy source (13). Adenine nucleotides [ATP, adenosine diphosphate (ADP), and adenosine (ADO)] function as signaling molecules (14) and modulate diverse signaling pathways through specific membrane receptors (15). ATP released through purinergic signaling activates both paracrine and autocrine signaling (16) and ATP hydrolysis further generates additional signaling molecules (17).

## Purinergic signaling

Purinergic signaling is a primitive evolutionary system (18), with short-term effects in neurotransmission and neuromodulation (19). Non-neuronal actions of purinergic signaling in mammals include immune response, pain, platelet aggregation, acute inflammation, exocrine and endocrine secretion, and endothelial-mediated vasodilatation (20). Trophic effects of purinergic signaling on cell proliferation, motility, and apoptosis mediate regeneration, wound healing, and cancer (21, 22).

**Figure 1:**
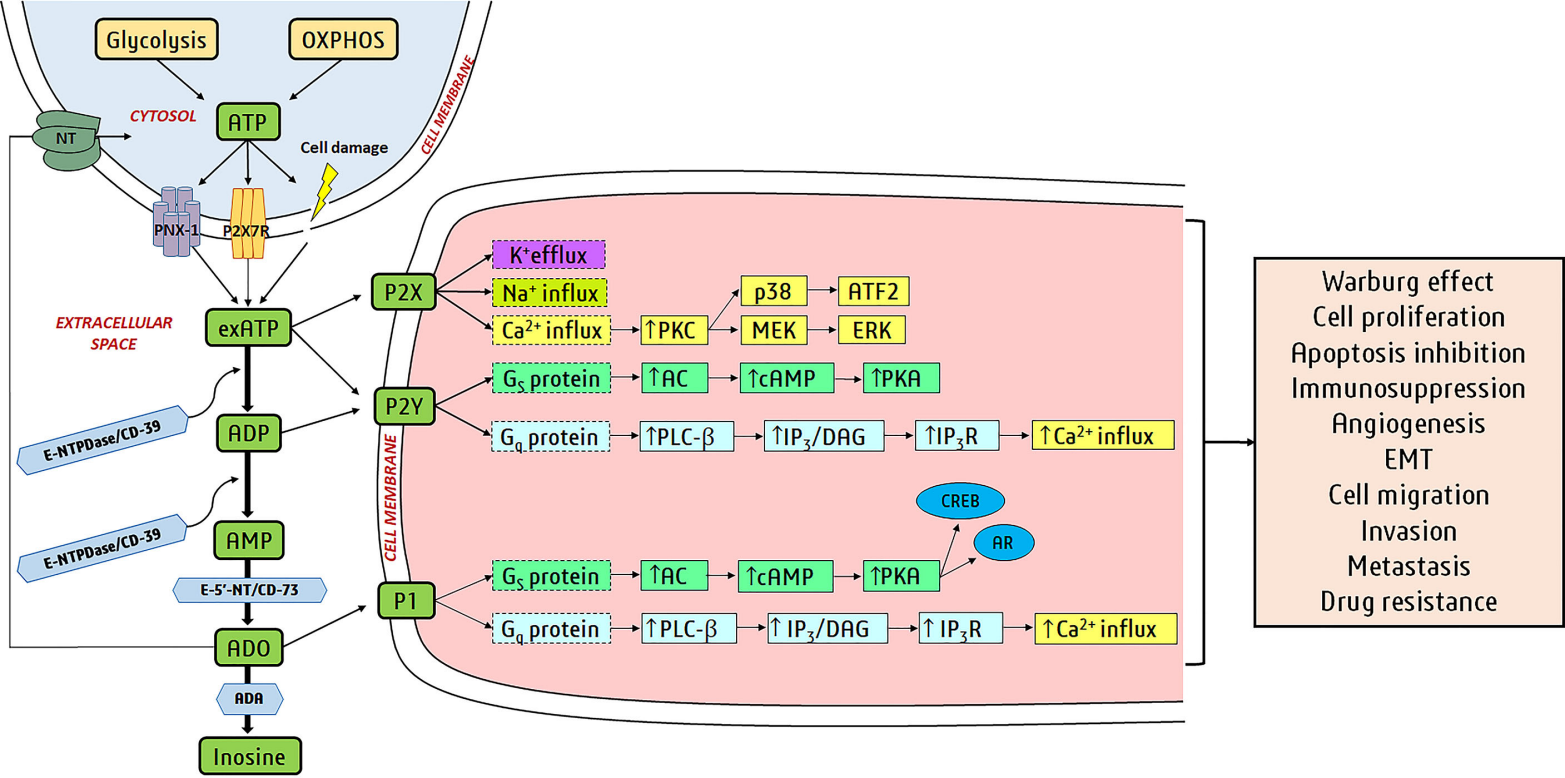
Purinergic signaling in cancer: Glycolysis and oxidative phosphorylation (OXPHOS) produce ATP in the cell, and it is released *via* cellular lysis, pannexin-1 (PNX-1) hemichannels, P2X7R, etc. to extracellular space. Cell membranes have several purine-hydrolyzing enzymes known as ectonucleotidases and include ectonucleotide triphosphate diphosphohydrolases (E-NTPDases/CD39), ectonucleotide pyrophosphatase/phosphodiesterases (E-NPPs), alkaline phosphatases (APs), and ecto-5’-nucleotidases (E-5’-NT/CD73). E-NTPDases hydrolyze exATP to ADP and also ADP to AMP. exATP activates ionotropic P2X receptors (ligand-activated ion channels) and metabotropic P2Y receptors (members of GPCR superfamily). AMP is hydrolyzed to ADO by E-5’-NT and APs. CD39 and CD73 hydrolyze exATP to ADP and AMP to ADO in cancer cells. ADP activates P2Y receptors and ADO activates P1 receptors (member of the GPCR family). ADO is hydrolyzed to inosine (Ino) by ADA or transported back into the cell by nucleoside transporters (NTs). The binding of purines to purinergic receptors turns on/off downstream signaling pathways. P1/P2Y receptor activation alters adenylate cyclase (AC) or phospholipase C-β (PLC-β) activity *via* different G proteins (G_s_ and G_q_ are stimulatory receptors). Activation of P2X receptors induces ion fluxes (Na^+^, K^+^, and Ca^2+^). All these actions alter the levels of secondary messengers like cAMP, inositol 1,4,5-trisphosphate (IP3), and Ca^2+^. IP3 binds to the IP3R receptors on the endoplasmic reticulum (ER), causing Ca^2+^ release from the ER. The secondary messengers regulate downstream proteins like protein kinase C (PKC), protein kinase A (PKA), cAMP response element-binding protein (CREB), androgen receptor (AR), mitogen-activated extracellular signal-regulated kinase (MEK), p38, extracellular signal-regulated kinases (ERK), and activating transcription factor 2 (ATF2). Transcription factors CREB, AR, and ATF2 induce gene expression for cancer development and progression.

Perivascular sympathetic nerves release ATP as a co-transmitter to cause smooth muscle contraction, whereas endothelial cells release ATP due to shear stress and hypoxia to produce nitric oxide and relaxation (23). Gentle mechanical distortion or hypoxia causes most cell types to release ATP physiologically (**Figure 1**) (24). Intracellular ATP is released *via* vesicular exocytosis, specific ATP binding cassette (ABC) transporters, connexin hemichannels, pannexin 1 (**Figure 1**), calcium homeostasis modulator (CALHM) channels, maxi-ion channels (MACs), volume-regulated ion channels (VRACs), and even purinergic (P2X7) receptors (**Figure 1**) (25, 26).

Extracellular ATP (exATP) either activates purinergic receptors or gets hydrolyzed by ectonucleotidases (E-NTPDases—ectonucleoside triphosphate diphosphohydrolases, CD-73-ecto-59-nucleotidase, ENPP—ectonucleotidepyrophosphatase/phosphodiesterase, AP—alkaline phosphatases, and ecto-5′-nucleotidase) (27–29). Ectonucleotidases thus limit ATP signaling and produce additional ligands for purinergic receptors like ADP to P2Y12 and adenosine to A2AR (A2-adenosine receptors) (30).

ATP has a minor role as a co-transmitter in a healthy human bladder; however, the purinergic component increases to 40% nearly during interstitial cystitis, neurogenic bladder, and outflow obstruction (20, 31). Purinergic signaling has crucial participation in trauma, ischemia, multiple sclerosis, amyotrophic lateral sclerosis, and Alzheimer’s, Parkinson’s, and Huntington’s diseases (25, 32). Purinergic signaling is also reported to be involved in mood disorders, epilepsy, and neuropsychiatric diseases (33, 34). Deregulation of purinergic signaling can lead to neurodegeneration, rheumatic immune diseases, inflammation, and cancer (34, 35). Thus, purinergic agents are being explored to treat disorders of the urinary tract (36, 37), gut (38), bone (39, 40), cardiovascular system (41), kidney (42, 43), reproductive system (44, 45), and cancer (46–51). Selective agonists and antagonists of purinergic receptors play a role in the development of thrombosis, stroke, osteoporosis, kidney failure, and colitis (20, 52, 53), which are orally bio-available and stable *in vivo*.

## Purinergic receptors

Purinergic receptors (purinoceptors) were first described as membrane receptors in 1976 (54), which mediate potent physiological actions of exATP on multiple cell types (55) including stem cells (56–58). Purinoceptors were primarily distinguished into two families—P1 and P2, where P1 mediates responses for adenosine (ADO) and P2 mediates responses for ATP/adenosine 5′-diphosphate (ADP) (59). P1 and P2 receptors mediate neurotransmission and neuromodulation in the central nervous system (CNS) and functions such as memory, feeding, locomotion, and cognition (60).

Early on, two subtypes of the P1 (adenosine) receptor were recognized (61, 62), followed by four subtypes using P1 (adenosine) receptors cloning and characterization (63). The P2 receptor was distinguished into two subtypes—P2X and P2Y—on a pharmacological basis in 1985 (64). In 1993, P2Y was cloned as the first G protein-coupled receptor (65, 66) and two P2X receptors were cloned as ligand-gated ion channels in 1994 (67, 68). Presently, the P2X receptor has seven and the P2Y receptor has eight recognized subtypes, and these receptors are sensitive to both purines and pyrimidines (15, 69, 70). Among the three purinergic receptors, i.e., P1, P2X, and P2Y, P1- and P2Y-mediated cell signaling events have opposite effects in biological systems (71). Almost every cell type expresses specific purinergic receptors and ectonucleotidases, and experiences continuous fluctuations of intracellular ATP (iATP), exATP, and ADO (72). A dissimilar set of purinoceptors and ectonucleotidases in cells generate diverse cellular responses, and a definite set of purinergic signaling components in a particular cell is known as a “purinome” (73).

## P1 receptors

Selective amplification and cloning were used to isolate complementary DNAs (cDNAs) for two P1 receptor subtypes (A1 and A2) in 1989 (74), followed by the A3 subtype (75). Currently, there are four P1 receptor subtypes—A1, A2A, A2B and A3 (63)—and A1 and A2A receptors have shown polymorphisms (76). P1 receptors are activated by ADO and belong to the G-protein-coupled receptor (GPCR) superfamily (77). P1 receptors are activated by extracellular adenosine (exADO) and AMP, and regulate adenylyl cyclase (AC) (78). A2A and A2B receptors couple to G_s_ proteins, stimulating increased levels of intracellular cAMP by AC activation, whereas A1 and A3 receptors negatively coupled to AC *via* G_i/o_ proteins and inhibit cAMP production (28, 70). Adenosine deaminase (ADA) hydrolyzes exADO and hence regulates ADO-associated signaling, and ADO is transported back into the cytosol by nucleoside transporters (NTs) (49). P1 receptor subtypes arbitrate physiological effects in the central nervous, cardiovascular, and immune systems (79, 80) and many P1-specific agonists and antagonists are identified and in use (**Table 1**) (133).

**Table 1:** Clinical trial status and cancer studies of purinoceptors agonists and antagonists.

Target receptor	Drug		Cancer type	Clinical trial phase	Recruitment status	Clinical trial number	Cancer studies
	Agonist	Antagonist					
A1	N^6^-Cyclopentyladenosine (CPA)		Nil	Nil	Nil	Nil	MCF-7 breast cancer cell line (81), renal cell carcinoma (82)
	R-isomer of N6 phenylisopropyladenosine (R-PIA)		Nil	Nil	Nil	Nil	Human tumor cell lines (83)
		1,3-Dipropyl-8-cyclopentylxanthine (DPCPX)	Nil	Nil	Nil	Nil	MCF-7 breast cancer cell line (81), renal cell carcinoma (82)
		CGS15943	Nil	Nil	Nil	Nil	Breast cancer (84)
		PSB36	Nil	Nil	Nil	Nil	Cisplatin-resistant ovarian cancer cells (85)
A2A	Regadenoson/CVT-3146		Breast cancer High-grade glioma	Nil Phase 1	Completed Active	NCT03505736 NCT03971734	
		KF17837	Nil	Nil	Nil	Nil	Human astroglioma cells (86)
		SCH58261	Nil	Nil	Nil	Nil	PC9 xenograft model (87)
		ZM241385	Nil	Nil	Nil	Nil	PC9 xenograft model (87)
A2B	Bay 60-6583		Nil	Nil	Nil	Nil	Human glioma and breast cancer cell lines (88), human epithelial lung cells (89)
		PSB-603	Nil	Nil	Nil	Nil	Colorectal cancer cells (90), head and neck squamous cell carcinoma cells (91)
		MRS1754	Nil	Nil	Nil	Nil	Renal cell carcinoma (92)
		PSB1115	Nil	Nil	Nil	Nil	Melanoma (93)
		Alloxazine	Nil	Nil	Nil	Nil	MCF-7 breast cancer cells (94)
A3	2-Cl-IB-MECA/Namodenoson/CF102		Advanced hepatocellular carcinoma Advanced hepatocellular carcinoma Hepatocellular carcinoma (HCC)	Phase III Phase I Phase II	Not yet recruiting Completed Completed	NCT05201404 NCT00790218 NCT02128958	
		MRS1191	Nil	Nil	Nil	Nil	Human small cell lung carcinoma (95)
		MRS1523	Nil	Nil	Nil	Nil	Melanoma cells (96)
		VUF5574	Nil	Nil	Nil	Nil	LNCaP and N1S1 cell lines (97)
		PSB10	Nil	Nil	Nil	Nil	Breast cancer (98)
		MRS1220	Nil	Nil	Nil	Nil	Glioblastoma stem-like cells (99)
P2X1	BzATP (benzoylbenzoyl-ATP, 2’(3’)-O-(4-benzoylbenzoyl)adenosine 5’-triphosphate)		Nil	Nil	Nil	Nil	Tumor-derived human endothelial cells (TEC) (100)
		NF449	Nil	Nil	Nil	Nil	Colorectal cancer cell line (101)
		NF023	Nil	Nil	Nil	Nil	Anticancer compound (102)
P2X2		PPADS (pyridoxal phosphate-6-azo(benzene-2,4-disulfonic acid))	Nil	Nil	Nil	Nil	Hepatocellular carcinoma (HCC) cell lines (103)
P2X3		AF353	Nil	Nil	Nil	Nil	Bone cancer pain (104)
		A317491	Nil	Nil	Nil	Nil	Cancer-induced bone pain (105)
P2X4	Ivermectin		Neoplasm	Phase II	Recruiting	NCT02366884	
	CTP (cytidine 5′-triphosphate)		Nil	Nil	Nil	Nil	Prostate cancer (106)
		5-BDBD	Nil	Nil	Nil	Nil	Prostate cancer (107)
		PSB-12062	Nil	Nil	Nil	Nil	Prostate cancer (107)
		PPADS	Nil	Nil	Nil	Nil	Human T47D breast cancer cells (108)
		Paroxetine	Nil	Nil	Nil	Nil	Gastric cancer cell AGS (109)
P2X7 (P2Z)	BzATP (benzoyl ATP)		Nil	Nil	Nil	Nil	Human cervical cancer cells (110), human HOS/MNNG osteosarcoma cells (111)
		KN62	Nil	Nil	Nil	Nil	Hepatoma cells (112)
		AZ10606120	Nil	Nil	Nil	Nil	Experimental neuroblastoma (113)
		A740003	Nil	Nil	Nil	Nil	Experimental neuroblastoma (113)
		A438079	Nil	Nil	Nil	Nil	Colorectal cancer (114)
		JNJ-47965567	Nil	Nil	Nil	Nil	Human cervical cancer cells (110)
		BBG	Nil	Nil	Nil	Nil	Human high-grade gliomas (115), glioma tumor (116)
P2Y1	MRS2365 ((N)-methanocarba-2MeSADP)		Nil	Nil	Nil	Nil	Prostate cancer (117)
	ADPβS (adenosine 5′-O-2-thiodiphosphate)		Nil	Nil	Nil	Nil	THP-1 leukemia cells (118)
	2-MeSADP (2-methylthioadenosine 5′-diphosphate)		Nil	Nil	Nil	Nil	Prostate cancer cells (119)
		MRS2179 (N6-methyl-2′-deoxyadenosine-3′,5′-bisphosphate)	Nil	Nil	Nil	Nil	Breast cancer (120)
P2Y2	2-thio-UTP		Nil	Nil	Nil	Nil	MCF-7 breast cancer cells (121)
	Ap4A (diadenosine 5′,5′′′-p1,p4-tetraphosphate)		Oral cancer	No phase	Unknown	NCT03529604	T47D and MCF7 breast cancer cells (122)
		Suramin	Prostate cancer Bladder cancer Brain and central nervous system tumors	Phase III Phase I Phase II	Completed Completed Completed	NCT00002723 NCT00006476 NCT00002639	
		AR-C118925	Nil	Nil	Nil	Nil	Head and neck squamous cell carcinoma cell lines (123)
P2Y6	MRS2693		Nil	Nil	Nil	Nil	Colorectal cancer (124)
	UDPβS		Nil	Nil	Nil	Nil	HeLa cell line (125)
		MRS2578	Nil	Nil	Nil	Nil	Breast cancer (126)
P2Y11	ATPγS (adenosine 5′-O-(3-thio)triphosphate)		Nil	Nil	Nil	Nil	Breast cancer (127)
	NF546		Nil	Nil	Nil	Nil	Hepatocellular carcinoma cells (128)
		NF157	Nil	Nil	Nil	Nil	Breast cancer cells (127)
		NF340	Nil	Nil	Nil	Nil	Breast cancer (129)
P2Y12	Clopidogrel		Head and neck Head and neck	Phase I Phase II	Recruiting Completed	NCT03245489 NCT00020189	
		2-MeSAMP (2-Methylthio-AMP)	Nil	Nil	Nil	Nil	MCF7 breast cancer cells (130)
P2Y14	UDP glucose		Nil	Nil	Nil	Nil	Lung cancer (131)
	UDP-galactose		Nil	Nil	Nil	Nil	Hepatocellular carcinoma (132)

## P2X receptors

P2X receptors are permeable ligand-gated cation channels (LGICs), where homotrimeric or heterotrimeric complexes of P2X1–P2X7 subunits form a trimer ion pore (69, 134, 135). P2X6R can only function as part of a heteromeric complex (136–140), whereas all other P2X subunits can assemble into homomeric or heteromeric ion channels. P2X1–7 receptors’ amino (N-) and carboxyl (C-) termini are intracellular and have binding motifs for protein kinases (69, 141, 142). The intracellular N- and C-termini of P2X receptors are linked to a large extracellular ligand-binding domain (with 10 conserved cysteine residues) using two transmembrane-spanning helices (TM1 and TM2) (143–145). TM1 assists with channel gating, while TM2 lines the ion pore (146).

Cloning of P2X-like receptors suggested ATP as an untimely evolutionary extracellular signaling molecule and a natural ligand for P2X receptors (147). ATP binds in the extracellular domain of P2X receptor and elicits a global conformational change that opens the channel for free cations’ movement in and out of the cell (148, 149).

Alternative splicing of the human P2X7R gene produced different isoforms with diverse cellular responses (150). P2X7R gene isoforms A and B are ubiquitously expressed as functional ion channels (151). P2X7A is a full-length variant, but the P2X7B isoform retains an intron between exons 10 and 11 with a stop codon, thus producing a shorter protein (152, 153). The truncated P2X7B lacks the C-terminal tail, which is crucial to macropore opening; however, P2X7B is still capable of opening the ion channel (153, 154).

P2X receptors mediate fast neurotransmission and at times are located pre-junctionally to promote the amplified release of co-transmitters, such as glutamate in primary afferent neuron terminals in the spinal cord (155). Activation of P2X receptors results in rapid membrane depolarization *via* Na^+^ and Ca^+2^ influx and K^+^ efflux (**Figure 1**) (156). P2XRs do not discriminate between Na^+^ and K^+^, but prefer the movement of Na^+^ down its electrochemical gradient at resting membrane potentials (157). P2XRs’ Ca^2+^ permeability is relative to Na^+^ (P_Ca_/P_Na_), but varies due to the subunit makeup of the functional channel (158). Thus, P2XR activation results in Na^+^-mediated plasma membrane depolarization and an increased free cytosolic Ca^2+^ concentration ([Ca^2+^]*i*), leading to action potential propagation and a myriad of Ca^2+^-sensitive processes like secretion (159, 160), muscle contraction (161, 162), and cell survival (163, 164). P2X1-, P2X2-, P2X3-, P2X4-, and P2X7-knockout mice and P2X1-overexpressing transgenic mice have multiple diseases associated with P2X receptor polymorphisms (165). Thus, selective P2X agonists and antagonists are available for multiple diseases like neurodegenerative diseases, thrombosis, stroke, osteoporosis, kidney failure, and cancer (20, 52, 53).

## P2Y receptors

P2Y are purinergic G protein-coupled receptors with eight accepted human subtypes: P2Y1, P2Y2, P2Y4, P2Y6, and P2Y11–14 (15, 166), and are stimulated by adenosine and uridine nucleotides and nucleotide sugars. The missing numbers for P2Y receptor correlate to non-mammalian orthologs with a little sequence homology to P2Y receptors, but with no functional proof of receptiveness to nucleotides (147). Based on sequence similarity, P2Y receptors have two distinct subgroups: G_q_-coupled receptors sharing a 28%–52% sequence similarity (P2Y1,2,4,6,11) and G_i_ */*_o_-coupled receptors with a 45%–50% sequence similarity (P2Y12,13,14) (15). Human P2Y1, P2Y12, and P2Y13 are activated by ADP, P2Y2 and P2Y11 are activated by ATP, P2Y2 and P2Y4 are activated by UTP, P2Y6 is activated by UDP, and P2Y14 is activated by UDP-glucose (167). For P2Y1 receptors, ADP is a stronger agonist than ATP (168).

P2Y1, P2Y2, P2Y4, and P2Y6 receptors are coupled to G_q_ proteins to activate phospholipase C (PLC), increase phosphoinositide level, and mobilize Ca^+2^ (169). ATP and UTP fully activate P2Y2 receptors; however, ADP and UDP are less effective as agonists (15). UTP is the strongest activator of the human P2Y4 receptor and P2Y6 receptors are UDP receptors (15).

P2Y11 receptor activation by ATP increases both cAMP and inositol triphosphate (IP3), whereas activation by UTP causes calcium mobilization without raising cAMP or IP3 (170). ATP is the preferred native ligand at P2Y11 receptors (170); however, ATPγS is a more efficacious agonist than ATP (15). P2Y12, P2Y13, and P2Y14 coupling to G_i_ proteins cause inhibition of adenylate cyclase (AC) (15). ADP is the original agonist of the P2Y12 receptor and the P2Y12 receptor activates RhoA and Rho kinase and phosphatidylinositol 3-kinase (PI3-K) *via* Gα_i_ (171). The P2Y13 receptor can couple to G_s_ at high ADP concentrations and to G_16_ and G_i_ simultaneously (172). Activation of several P2Y receptors stimulates mitogen-activated protein kinase (MAPK), especially extracellular signal-regulated protein kinase 1/2 (ERK1/2) (15).

## Purinergic signaling in cancer

Purines perform autocrine–paracrine functions in cancer cells by ATP production and release, which causes cell proliferation, tumor growth, and immunosuppression (72). Cancer cells use aerobic glycolysis to synthesize ATP (Warburg effect), lactate production, and consequent extracellular acidification (72). Cancer cells release ATP from pannexin-1 (PANX1) hemichannels, P2X7R, or by cellular lysis and can activate either P2 receptor (**Figure 1**) (72). ExATP activates P2 receptors in cancer cells (primarily P2X7R and P2Y2R) *via* an autocrine–paracrine way to encourage proliferation, epithelial-to-mesenchymal transition (EMT), and migration (72). Ectonucleotidases (CD39 and CD73) hydrolyze exATP to ADO and increased ADO inhibits the anti-tumor function of innate immune cells and effector T cells (T_eff_, CD4+ and CD8+), and monocyte differentiation into associated tumor macrophages 2s (72).

Purines in the tumor microenvironment (TME) have gained immense attention, as high concentrations of ATP and ADO in the TME are established biochemical markers of cancer (173, 174). Treatment of pheochromocytoma PC-12 cells with maitotoxin released ATP and activated purinergic receptors (175); mechanical stimulation of Ehrlich ascites tumor cells released ATP (176) and TGF-β stimulation of A549 human lung cancer cells released ATP by exocytosis (177). *In vivo* ATP monitoring in a tumor-bearing mouse using reporter cells carrying an exATP sensor revealed that exATP is a primary source of exADO in the TME (178). ExADO is copious in microdialysates from tumoral core regions (178) and tumor hypoxia promotes ADO formation by hypoxia-inducible factor 1α/β (HIF-1α/β)-induced CD39 and CD73 expression (179–182). Ectonucleotidases CD39 and CD73 are immune checkpoints in cancer, expressed in cancer cell lines, stromal cells, and immune cells, and modulate ATP and ADO levels in the TME (183). ATP is released through the PANX1 channel and a truncated PANX1 protein (PANX1^1-89^) is overexpressed in metastatic human cancer cell lines (184). PANX1^1-89^ along with wild-type PANX-1 promotes gain of function of channel activity, an increased ATP release, and resistance of cancer cells to mechanical deformation (184). ATP is also released through P2X7R and P2X7R association with NLRP3 inflammasome producing pro-inflammatory cytokines, like IL-1β and IL-18 (185–187). Tissue damage and cell death induced during chemotherapy/radiotherapy produce damage-associated molecular patterns (DAMPs), which are mostly ATP (188). The CD39/CD73 pathway quickly converts this exATP into ADO, and thus, a particular equilibrium of purines in the TME decides the success of a given clinical treatment (189).

P2X7R is the most studied purinergic signaling element for its role as an apoptotic inducer (190). However, P2X7R promoted proliferation in lymphoid cells (191), instead of its previously designated role as a pro-apoptotic and necrotic cationic channel receptor (192). Elevated exATP levels and activated P2X7R had growth-promoting effects *via* MAPK/ERK kinases and stimulated *de novo* synthesis of pyrimidine nucleotides (193). P2X7R has a pro-mitotic function in B-cell chronic lymphocytic leukemia, and higher P2X7R expression was seen in patients with an aggressive form of chronic lymphocytic leukemia (194). P2X7R expression promoted prostate and breast tumor progression (195); thus, P2X7R has a dual ability to induce apoptosis and promote cell survival (196).

Increased P2X7R expression is found in breast (195), thyroid (197), mesothelioma (198), ovarian (199), pancreatic (200), colon (201, 202), osteosarcoma cell (111), and liver (203) cancer, and higher P2X7R expression associated with a high tumor grade. Elevated P2X7R expression was originally known to induce apoptosis; however, proliferating and/or survival effects of P2X7 were reported later on (204). P2X7R as a growth-promoting receptor was supported by its exogenic expression in numerous cancer cell lines and tissues, by high amounts of ATP in the TME, and by positively regulating aerobic glycolysis (204). P2X7R stimulates ERK phosphorylation-dependent and -independent intracellular Ca^2+^ rise (205, 206), the PI3K/AKT/GSK3β/β-catenin pathway, and mTOR/HIF1α/VEGF signaling (199, 207).

P2X7B is a widely studied splice variant of P2X7 in cancer due to its pro-tumoral role and powerful constructive effect on P2X7A (208, 209). Removal of the lengthy carboxy-terminal in P2X7B enables its growth-promoting potential in response to ATP; however, it loses its cytotoxicity linked to the pore formation (208). P2X7B promoted ATP secretion, activated NFATc1 (nuclear factor of activated T cells c1) proliferative pathway, and supported soft agar invasion (208, 209). The role of P2X7B in solid cancer was first reported in human osteosarcoma, where P2X7 isoforms were expressed in osteosarcoma tissues and P2X7B expression was the most prevalent (209). Transfection of Te85 osteosarcoma cells with P2X7B increased proliferation and reduced bone deposition in serum-starving conditions compared to non-transfected cells (209). Bradykinin treatment of human neuroblastoma cell lines (CHP-100, SH-SY5Y) upregulated P2X7B and cell proliferation, suggesting a growth-metastatic stimulus by P2X7B overexpression (210). The P2X7B isoform favored metastasis and resistance in tumor cells (208, 209, 211), caused malignancy of neuroblastoma (NB) cells (210), suppressed autophagy, and induced drug efflux and EMT in NB (212).

P2Y receptor overexpression also promotes cell proliferation and is observed in basal cell and squamous cell carcinoma biopsies (213). cDNA microarray analysis showed higher expression of P2RY2 transcript in gastric cancer biopsies than adjacent healthy tissue (214). P2Y2R promoted cell proliferation in C6 glioma cells *via* the Ras/Raf/MEK-1 pathway, which was modulated by PLC/PKC and Ca^2+^ (215).

ADO is the chief product from exATP, and ADO receptor A2BR functions as a cell proliferation promoter in human hepatocellular carcinoma (216), prostate cancer (217), colorectal carcinoma (218), breast cancer (219), oral squamous carcinoma (220), head and neck squamous cell carcinoma (HNSCC) (91), and bladder urothelial carcinoma (221). Pharmacological/genetic inhibition of A2BR decreased cell proliferation in colon carcinoma cells, human oral cancer, and bladder urothelial carcinoma (218, 220, 221).

Contrary to P2Y2R activity, P2X4R expression inhibited proliferation in gastric cancer cell lines (222) and reversed P2X7R-induced proliferation in breast cancers (223). P2X7R is downregulated in endometrial cancer and P2X7R activation-induced apoptosis in endometrial carcinoma cells (224) and inhibited virus-induced skin cancer formation *in vivo* (225). P2Y2 receptor activation suppressed cell proliferation in endometrial carcinoma cells (HEC-1A and Ishikawa) (226), human esophageal cancer cells (227), human colorectal carcinoma cells (HT29 and Colo320 D) (228), and nasopharyngeal carcinoma cells (229). P2Y6R activity inhibited cell proliferation through store-operated Ca^2+^ entry (SOCE) and β-catenin (230). Consequently, purinergic signaling positively regulates cell proliferation; however, functional interactions among purinergic receptor subtypes determine the ultimate effect of purinergic signaling in cancer.

## Warburg effect during purinergic signaling

HEK293 and ACN (neuroblastoma) cell lines transfected with P2X7R showed an amplified lactate production and cell proliferation, signifying aerobic glycolysis and the Warburg effect (231). P2X7R expression upregulated glycolytic promoters like glucose transporter Glut1, enhanced intracellular glycogen stores, repressed pyruvate dehydrogenase (PDH) activity, and increased protein kinase B (PKB/Akt) phosphorylation and hypoxia-inducible factor 1α (HIF-1α) expression to evade aerobic adaptations (231).

P2X7R accomplishes Warburg and proliferative effect by functioning as an ion channel and a highly conductive non-selective pore (232). In this way, P2X7R stimulated a rise in mitochondria’s resting membrane potential and basal calcium levels (232). P2X1R and P2X7Rs activation in leukemia T cells (Jurkat) caused higher intracellular calcium levels and uncontrolled proliferation, and pharmacological inhibition of these receptors decreased cell proliferation, mitochondrial activity, and calcium signaling (233).

A549 (human non-small cell lung cancer cell line) internalized exATP by clathrin-mediated endocytosis (CME), caveolae-mediated endocytosis, and macropinocytosis (234), which served as glycolytic ATP in cancer cells (235) and favors proliferation, drug resistance, and EMT (234). High exATP and exADO concentrations are typical of neoplastic cells, thus recognizing ADO as a pro-tumoral factor (236). Toll-like receptor 3 (TLR3) is a pro-inflammatory receptor and promoted glucose consumption and lactate release in prostate cancer cell lines *via* HIF-1α and extracellular activation of A2BR (237). ZM241385 and SCH5826 are A2AR antagonists and repressed proliferation in cancer-associated fibroblasts and human tumor xenografts in mice (87).

## Purinergic signaling in cell proliferation and death

Continual proliferation characterizes tumor cells and growth factors stimulate cell proliferation through receptors with intracellular tyrosine kinase domains (238). ATP is not a member of the growth factor family and P2RX7 has no tyrosine kinase domains; however, low concentration of ATP activates P2RX7, cell proliferation (111, 200, 239–241), and kinase activity (242). P2RX7 activated cellular sarcoma tyrosine kinase (c-Src), PI3-K/Akt, MAPKs (ERK1/2, p38, and JNK), and protein kinase C (PKC) (243). P2RX7 activated these kinases in response to ATP, indirectly *via* Ca^2+^ or its co-localization with kinases in lipid rafts using a lipid-interaction motif in its C-terminus (amino acids from 574 to 589) (244–247). Reduced P2RX7 expression resulted in the development of various cancers (150, 248–250) and P2RX7 inhibition by shRNA in glioma tissue increased EGF and phosphor-EGF protein expression (251). P2RX7 is not known to inhibit tumor suppressor proteins like TP53 or retinoblastoma-associated (RB), but a death domain is localized in its C-terminus (244).

The ATP/P2RX7 axis can induce or inhibit apoptosis. The ATP/P2RX7 axis promoted p53 protein levels, apoptosis, and necrosis in mesangial (252) and human cervical cancer cells (253), and induced apoptosis in acute myeloid leukemia (AML) (254). Tumors grew faster in *p2rx7* ^−/−^ mice (255, 256), and P2RX7 inhibition induced apoptosis in colorectal cancer cells (241) and the MCF7 breast cancer model (257). P2RX7-induced necrosis released growth factors to advance the proliferation and invasiveness of cancer cells (200) and immunomodulatory cytokines. A non-conformal receptor (nfP2RX7) promotes tumor growth, has calcium channel activity, but is incapable of inducing cell death (258, 259).

## Purinergic signaling in angiogenesis

Tumors facilitate neoangiogenesis, i.e., growth of novel blood vessels from a prior vasculature for oxygen and nutrients (260). Tumor vasculature is abnormal with hardly any perivascular cells, and this brings hypoxia and acidity in TME, which facilitates tumor aggression (261). Vascular endothelial growth factor (VEGF) couples with VEGF receptor 2 (VEGFR2) to boost endothelial cell propagation (262). P2RX7 promoted VEGF discharge in human monocytes (263, 264) and P2RX7-expressing tumor xenografts of human embryonic kidney cells were found to be more angiogenic than the control cell tumors (239). The ATP/P2RX7 axis stimulates PI3K/Akt and NF-κB pathways to induce VEGF and higher vessel density (113, 239, 265).

## Purinergic signaling in migration and metastasis

Malignant cells invade surrounding and distant tissues, and this metastatic extension of the primary tumor is responsible for approximately 90% of mortality in cancer patients (266). Metastatic stages in a primary tumor involve cell–cell adhesion loss, EMT, anoikis evasion, migration, invasion, mesenchymal–epithelial transition (MET), and embedding as a secondary tumor (267). EMT initiates metastatic stages, as epithelial cells morph into a mesenchymal phenotype with a superior invasive and migration capacity (268). Transforming growth factor-β (TFG-β), Wingless/Int (WNT), and epidermal growth factor (EGF) stimulates EMT promoter transcription factors, i.e., TWIST and SNAIL (269). TWIST and SNAIL cause loss of epithelial marker expression [E-cadherin, zona occludens-1 (ZO-1), and keratins] and induce expression of mesenchymal markers [N-cadherin, metalloproteinases (MMPs), and vimentin], thus promoting metastasis (270).

ATP is the cellular energy currency, but during purinergic signaling, it engages in EMT, migration/invasion, and metastasis in various cancer types (271). High ATP concentrations in lung cancer cell lines favored cell detachment, migration, and invasion, with increased MMPs, vimentin, SNAIL and SLUG expression, and filopodia development and cell protrusions (235). A decrease in E-cadherin and ZO-1 expression by the micropinocytosis of exATP and genetic deletion of sorting nexin-5 (SNX5, a gene involved in intracellular trafficking) reduced cancer cell proliferation and metastasis (235). TGF-β1 elicited lung cancer cells to release ATP and activated P2 receptors (177). TGF-β1 induced remodeling of actin and cell migration by autocrine stimulation of P2X7R and knockdown or pharmacological inhibition of P2X7R suppressed these processes (177). A mutated EGFR in PC9 (human lung adenocarcinoma cell line) caused a constitutive activation of P2X7R and cell migration, even without TGF-β1 (272). AG1478 is an EGFR inhibitor and abolished PC9 cells’ motility and lamellipodium extension, showing a TGF-β1/P2X7R/EGFR cross-signaling during cell migration (272).

P2X7R promoted metastasis in pulmonary (177, 269), prostatic (273), mammary (108), pancreatic (200), glioma (274), osteosarcoma (111), and glioblastoma stem cell (GSC) cancer (275). P2RX7 high expression in MDA-MB-435 cells (an invasive human breast cancer cell line) activated pro-migratory phenotype and invasiveness by releasing extracellular matrix-degrading proteases *in vitro* and *in vivo* (276, 277). The ATP/P2X7R axis encouraged an autocrine-mediated TGF-β-instigated cell migration in lung cancer cells (177). In response to ATP, P2X7R promoted cell migration and invasion in pancreatic ductal adenocarcinoma (200), human breast cancer, and colon cancer cells, where the ATP-activated PI3K/Akt/GSK3 pathway mediated E-cadherin expression and EMT (108, 241). In a mammary 4T1 cancer mouse model, P2RX7 expression promoted tumor growth and metastasis and was reversed by P2X7R antagonists (108). Tumors with distant metastasis showed higher expression of P2X7R in broncho-alveolar lavage (278) and more lung tumors were observed in *p2rx7* ^−/−^ mice injected with B16 melanoma cells, suggesting that endogenous P2X7R activity hinders B16 migration and invasion (256). Bradykinin induced P2X7B expression and promoted neuroblastoma metastasis in bone marrow using high exATP levels in the bone marrow as growth, seeding, and anti-apoptosis stimulus (210).

In most breast cancer cell lines (121, 279, 280), the invasive edge of the breast tumor tissue and tumor embolus in lymphatic sinuses express P2Y2R highly, proving the participation of P2Y2R in metastasis (279). Breast cancer cell lines with more exATP are highly metastatic (281), and migration and invasion are moderated through MEK/ERK1/2-dependent signaling pathway activation (121, 279, 282) and ATP-P2Y2R-β-catenin (280). Activation of P2Y2R increases EMT-related gene expression and transactivates a pathway amid P2Y2R and EGFR in ovarian (283) and prostate cancer cells (284).

A1R antagonist 1,3-dipropyl-8-cyclopentylxanthine (DPCPX) reduced proliferation and migration in renal cancer cell (RCC) lines, and tumor growth *in vivo*, while A1 agonist N^6^-Cyclopentyladenosine (CPA) rescued RCC cell migration (82). Higher CD73 expression was seen in lymph nodes of HNSCC patients, and CD73 encouraged HNSCC migration and invasion *in vitro* by A3R activation *via* the EGF/EGFR signaling pathway (285). CD73 expression promoted mesenchymal phenotype in hepatocellular carcinoma and A2AR activation restored the effect of CD73 knockdown, suggesting an adjuvant treatment of CD73 and A2AR inhibitors (286). Low ADO concentrations reduced migration and the invasive capacity of prostate and breast cancer cell lines (287) and human cervical and ovarian cancer cell lines (288, 289), but enhanced stemness and EMT gene expression in gastric cancer cells by activation of A2AR and the Akt/mTOR pathway (290).

A2BR activation induced a partial EMT through cAMP/PKA and MAPK/ERK transduction pathways in human type II alveolar epithelial cells (A549) (89) and BAY 60-6583 (selective A2BR agonist) counteracted TFG-β-induced EMT in A549 cells (89). ADO augmented migration *via* the A2BR/AC/PKA/cAMP axis in MDA-MB231 breast cancer cells (219). A2BR antagonist PSB-603 inhibited cell proliferation and transmigration in HNSCC-derived cell lines and MRS1754 (a selective A2BR antagonist) reduced proliferation and migration in RCC 769-P and Caki-1 cell lines (91, 92).

A3R activation detained cancer cell motility and invasive capacity by hampering NADPH oxidase activity and AC/PKA in AT6.1 rat prostate (291). A3R obstruction reduced cell migration and invasion in glioblastoma (GBM) patient-derived primary cultures of stem-like cells and a GBM cell line (292). Collagen architecture modulates ATP : ADP ratio, and this ratio increases in dense extracellular matrices (ECMs) where migration is arrested and decreases in aligned ECMs where migration is eased (293). Thus, the adhesion environment changes the cellular energy necessity, and ATP increases in the ECM assist metastasis (293). ATP is also vital during invadopodia formation, and even in the absence of MMPs, it helps in F-actin network growth (294).

## Purinergic signaling in anti-tumor immune response

The immune system fights infections and immune surveillance eliminates cancer cells *via* checkpoint inhibitor (CPI) expression, immunosuppressive factor secretion (295, 296), and T_eff_ cell and natural killer (NK) cells (297). These immune responses against cancer cells are dulled by immune-suppressive cells [regulatory T (Treg) and myeloid-derived suppressor cells (MDSCs)] (298).

Ectonucleotidases mediate ADO accumulation, where CD39/NTPDase1 converts ATP to ADP/AMP and CD73 converts AMP to ADO (299). Tumor cells with activated CD73 favor cell adhesion through EGF and ADO accumulation energizes cell proliferation and metastasis by inducing intracellular cAMP’s downstream signaling through A2A and A2B, leading to immunosuppression (300). exATP evokes a “find me” signal evoking an immune response, and this response is overturned by exATP conversion to ADO by CD39 and CD73 (183, 189, 301). ADO, thus, has an immunosuppressive role and dismantles the anti-tumor immune attack in the TME (183, 189, 301). Conditions like hypoxia, necrosis, and inflammation prime cells to release DAMPs, mainly ATP (187), and exATP employs macrophages, neutrophils, and dendritic cells (DCs) to resolve cellular damage (302–304). Chemotherapeutic agents in AML induced exATP and promoted the upregulation of Treg cells (305), suggesting that ATP mediates the communication of tumor cells and immune system components and modulates the inflammatory response in the TME. Post-anti-cancer therapies, dying cancer cells release ATP and activate P2X7R in DCs, leading to IL-1β secretion *via* P2X7R-dependent NLRP-3 inflammasome assembly (306, 307), and this suggests the role of NLRP-3 inflammasome in the efficacy of anti-cancer therapies. Hypoxia in cancer cells favors ADO accumulation and CD39 and CD73 overexpression in the TME, by HIF-1 transcription factor activity (179–182).

Ovarian cancer cell lines generated ADO to attract myeloid cells and induce their differentiation to macrophages with a non-inflammatory phenotype (M2-TAM) with CD39 overexpression (308). CD39-expressing TAMs and CD73-expressing stromal fibroblasts collaborated to amplify ADO levels and immune-suppressive effects in ovarian cancer cell lines (308). ADO buildup is deadly for immune surveillance, and CD39/CD73-targeted antibodies promoted immunosuppression in macrophages, DCs, and T cells (309), making these ectonucleotidases the obvious immunity-strengthening targets. Antibodies against CD39 and CD73 improved NK and T-cell cytotoxicity in ovarian cancer cell lines by ADO reduction (310). CD73 expression and ADO levels increase post-focal radiotherapy, and thus, blocking CD73 during focal radiotherapy enhanced DC infiltration and anti-tumor T-cell-dependent responses in breast cancer cells (311).

P2RX7 has an extensive role in immune cells and tumor onset (312). P2RX7 is expressed in human macrophages and DCs to alter cytokine production owing to exATP (313–316). The ATP/P2RX7 axis boosts anti-tumor immune response, as exATP attracts DC precursors to the tumor bed, in the immediacy of dying cells, and promotes their ability to present tumor-associated antigens (317). P2RX7 activation in macrophages and DCs releases NLRP3 to increase IL-1β and IL-18 production and NLRP3 has pro- and anti-tumorigenic roles in cancers based on cytokine quantity (318). AML patients have upregulated *P2X7R* receptor mRNAs (splice variants A and B) in AML blasts, suggesting *P2X7RA* and *P2X7RB* as potential prognostic markers in AML patients (211, 319). Murine B16 melanoma cells express high levels of P2X7R, and its xenotransplantation in wild-type and *p2rx7* ^–/–^ null mice showed accelerated tumor growth in the *p2rx7* ^–/–^mice (320). Compared to wild-type animals, *p2rx7* ^–/–^ mice tumors showed immunosuppression with a higher number of T_reg_ cells and fitness markers (OX40, PD-1, and CD73) and a declined number of T_eff_ and CD8^+^ T cells (320). Ectonucleotidase CD73 was expressed highly in macrophages, T_reg_, and CD8^+^ T_eff_, while CD39 was prominent in T_eff_ in tumor-bearing *p2rx7* ^–/–^ mice (320). These CD39/CD73 expression alterations reduced exATP and increased ADO in the TME, causing immunosuppression in *p2rx7* ^–/–^ mice tumors (320). Blocking P2X7 by using A740003 in wild-type mice reduced the growth of tumors, improved the immune response, elevated IFN-γ, and reduced IL-1β levels, without changing exATP levels (320). Thus, P2X7R is a key determinant of anti-tumor immune response due to its effects on immune cell infiltration (320). Leukocyte common antigen (CD45+)-immune cells in the lung adenocarcinoma patients’ TME had lesser P2X7R pore function than CD45+ cells from outside the tumors, suggesting higher P2X7B mRNA levels in the immune cells from the TME than normal lung tissue (321).

P2X7 stimulation restricted tumor suppression by inducing stress-induced premature senescence (SIPS) in tumor-infiltrating lymphocytes (TIL) and transfer of *p2rx7^-/-^* CD8 T cells reduced tumor growth with improved survival in lymphopenic mice (322). However, in a mouse melanoma CD8^+^ T-cell adoptive transfer model, the number of tumor-specific *p2rx7^-/-^* CD8^+^ T declined with an increase in tumor burden, and this was concurrent with decreased proliferation and higher apoptosis (323). *In vitro* stimulation of P2RX7 *via* ATP analog BzATP controlled B16 melanoma by CD8^+^ T cells, suggesting that exATP sensing on CD8^+^ T cells by P2RX7 diminished melanoma tumors (323). This presents P2X7 as a purinergic checkpoint that may be targeted to enhance anti-tumor response.

A2AR is the foremost P1 causing immunosuppression and its genetic deletion eased tumor eradication by T cells (324). Supplemental oxygenation (hyperoxia) helped CD8+ T cells to infiltrate tumors, regress the tumor, weaken immunosuppression by Treg, and enhance chemokines and cytokine levels, through the hypoxia/ADO/A2AR pathway, and these effects were not shown by A2AR^-/-^ mice (181). A2AR antagonist co-administration improved the efficiency of PD-1 antibodies (325) in reversing the immunosuppression of induced tumors, thus making A2AR an immunotherapy target (326). Tissues lacking A2AR showed improved tumor rejection, diminished immunosuppression, and enhanced IFN-γ secretion by T cells (327). A2AR-mediated ADO signaling ablation matured NK cells, improved anti-tumor immunity, and delayed tumor instigation and growth (328). A2BR works synergically with A2AR in colorectal cancer cells to participate in the NT5E/ADO-dependent immune checkpoint to launch immunosuppression (329). A1R deletion reduced human melanoma cell line and tumor growth in immune-deficient xenografts, upregulated PD-L1 levels, and inactivated co-cultured T cells, which compromised anti-tumor immunity *in vivo* (330), indicating the critical role of ARs in the anti-tumor immune response.

## Purinergic signaling in cancer stem cells

Cancer stem cells (CSCs) within the tumor are related to cancer metastasis, chemotherapeutic drug resistance, and tumor recurrence (331). CSCs drive tumor spread and preservation due to their stem cell-like properties (332) of self-renewal and multi-lineage differentiation potential (333). Unlike normal tissue renewal, CSCs do not mature and form a core in a tumor to sustain tumor growth (334). CSCs express explicit cell surface markers like CD166+, CD133+, CD44+, CD29+, and CD24+ (335), and the nature and expression level of these markers vary depending on the type of cancer (336). Endogenous ATP release caused a proliferation in various human glioma cell lines (U251MG, U87MG, and U138MG) *via* both P1 and P2 receptor signaling (337). ATP treatment decreased the number and size of tumor spheres in human U87 or U343 and rat C6 gliomas (338), by upregulating P2X4, P2Y1, and P2Y14 in tumor spheres (338) and reduced the number of Nanog, Oct-4, and CD133+ CSCs (338). Thus, ATP and purinergic receptors are potential pharmacological targets for CSC therapy (338, 339).

Expression of the P2X7 receptor correlates with the degree of cell differentiation and its higher expression and activity are seen in embryonic stem cells (340). A decrease in pluripotency markers suppresses P2X7 receptor expression and activity, and differentiation was induced in quiescent cells upon P2X7 inhibition pharmacologically (340, 341). Sustained P2X7 stimulation in glioblastoma multiforme stem-like cells from primary human tumors decreased CSC proliferation due to cell death and growth arrest (342). This growth arrest induced a quiescent state in the surviving cells and caused cell regrowth and sphere formation 2 weeks after the end of the treatment (342), suggesting P2X7-induced chemoresistance and tumor recurrence.

P2X7B promoted osteosarcoma by a higher expression in osteoblast precursor cells than differentiated osteoblasts (343). ATP analog and P2X7 agonist 2’[3’]-O-[4-benzoylbenzoyl]-ATP (BzATP) increased the expression of P2X7A and B in human GSCs and supported GSC invasion (275). P2X7 antagonist A438079 nullified BzATP effects on P2X7A and P2X7B expression (275).

## Purinergic signaling in drug resistance

Dying tumor cells release ATP, making exATP levels multiple times higher in cancer tissues than the analogous normal tissues (178). exATP induces cellular stress and anti-cancer therapy resistance *via* inflammation, hypoxia, and platelet aggregation (344). Anti-cancer drugs (targeted and chemotherapeutic) increase iATP and cancer cell survival by exATP (345). Sunitinib promoted drug resistance in NSCLC A549 cells by internalizing exATP through CME, caveolae-mediated endocytosis, and macropinocytosis, which elevated iATP levels to 150%–200% of the original iATP concentration (234, 345, 346). Increased iATP levels by exATP is not a momentary effect and the elevation lasts till exATP is present in the TME (346).

Macropinocytosis activation is a hallmark of oncogenic Ras mutations harboring cancers, and macropinocytosis/endocytosis-mediated elevated iATP levels from exATP internalization promote drug resistance (347). p21-activated kinase 1 (PAK1) is an essential enzyme for macropinocytosis, and its inhibition by siRNA knockdown or IPA3 inhibitor resulted in lower iATP levels and survival in exATP- and sunitinib-treated A549 cells (345). Abundant iATP molecules after exATP internalization contend for the ATP binding site on receptor tyrosine kinases (RTKs) with tyrosine kinase inhibitors, inducing phosphorylation and, in turn, activation of downstream signaling events causing drug resistance (345).

Internalized exATP molecules served as an energy supplement, regulated ABC transporter expression levels, and increased cell survival in A549 lung cancer and SK-Hep-1 cells (345), indicating intense effects of exATP on transporter activity of ABC to enhance anti-cancer drug efflux and drug resistance. exATP hastened EMT, mobility, and invasion, increased MMP expression, increased levels of EMT-transcription factors (vimentin, Snail, and Slug), and decreased epithelial markers in lung cancer cells (235). These effects were TGF-β-independent and partially dependent on the purinergic signaling (P2X7) activation (235), suggesting exATP-induced drug resistance *via* EMT. exATP promotes drug resistance through P2 receptors as an extracellular messenger (72), and hence, the P2X and P2Y receptor families have emerged as potential candidates for chemotherapy resistance (348, 349). ENTPD1/CD39 convert exATP to AMP and activate cAMP-mediated mitochondrial stress response *via* the CD39/P2RY13/cAMP/PKA axis and aided resistance to cytarabine in AML, proposing exATP and CD39 as key factors in AML chemoresistance (348).

Activated platelets in tumor cells release ADP in the extracellular compartment, and ADP-activated P2Y12 in pancreatic ductal adenocarcinoma (PDAC) increased expression of markers for gemcitabine resistance like human equilibrative nucleoside transporter 1 (hENT1) and cytidine deaminase (CDD) (350). P2Y12 inhibition by ticagrelor blocked the survival of PDAC cells induced by platelet-derived ADP and ATP (350).

P2X7 receptor is bi-functional, and depending on the cell type and level of activation, it can trigger either cell growth or death (191, 194, 351–353). P2X7 overstimulation by mM ATP concentration formed pores and caused cell death in rat peritoneal cells (RPCs) and in P2X7 transfected 1321N1 cells (1321rP2X (7)-11) (354). Low-level stimulation of P2X7 endowed P2X7-expressing cells with proliferative advantage by enhancing aerobic glycolysis, OXPHOS, and biosynthesis (173). P2X7 expression helps cancer cells to invade and metastasize (153, 173), and thus, its antagonists inhibited tumor growth and migration (344). Non-pore functional P2X7 (nfP2X7) is a distinct P2X7 conformation that cannot form a functional pore upon exATP stimulation (259). ATP concentration equivalent to TME drives nfP2X_7_ expression and survival in tumor cells (259), suggesting a role of nfP2X7 in promoting resistance to apoptosis and chemotherapies.

Some chemotherapeutic agents incite immunogenic cell death (ICD), leading to immunological memory generation and improved relapse-free survival (355). Stressed/dying tumor cells release exATP as a DAMP during ICD (259), and thus, ICD depends on exATP release as well as on its stability in the TME (356). exATP release drives DC recruitment and activation by P2RX7 ligation, generating adaptive immunity against cancer by tumor-derived antigens and IL-1β secretion (306).

P2X7B caused daunorubicin and radiotherapy resistance in P2X7B-overexpressing cells by increasing exATP levels in the TME (211, 357). P2X7B expression increased neuroblastoma chemoresistance through drug efflux *via* multi-drug resistance protein (MRP)-type transporters, suppressing autophagy, promoting EMT and resistance to retinoids, and retaining stem-like phenotype (212).

exATP communicates with immune cells like monocytes, T cells, B cells, macrophages, eosinophils, and neutrophils to execute immune-activating functions (306). exATP is converted to ADO by CD39 and NT5E/CD73, and while exATP promotes anti-tumor immunity, ADO attenuates it (349, 358). CD39 and CD73 have emerged as potential targets for anti-cancer immunotherapy, as these limit exATP conversion to ADO (355). CD39 blockade augmented exATP/P2X7-mediated pro-inflammatory anti-tumor response and release of IL-18, which facilitated increased infiltration of intra-tumor T_eff_ cells and overturned anti-PD-1 resistance (349). Adenosine-induced signaling diminishes the anti-tumor action of immune cells (macrophages, T cells, B cells, DCs, mast cells, and NKs) and activates T_reg_ cells, creating an immune-suppressive environment to diminish immune-therapy efficacy (344, 359). Tumor cell autophagy is critical for chemotherapy-induced ICD, and it promotes exATP release over adenosine generation (360); thus, purinergic signaling inhibition might improve anti-cancer drug response.

Fe^3+^ and doxorubicin-releasing nanoparticles depleted exATP *via* metal ion-triphosphate coordination and sensitized chemotherapy (361), suggesting that exATP depletion may also reduce drug resistance. Macropinocytosis inhibition reduced cellular uptake of exATP and iATP levels, alleviating EMT and drug resistance promoted by exATP (361), suggesting a multifunctional role of ATP as an energy currency, a drug efflux facilitator, and a signal transducer for cell survival and tumor migration/invasion.

## Conclusion

Cancer is a complex disease and self-regulation of differentiation, growth, and expansion has allowed cancer cells to succeed biologically over normal cells (266, 362). This review summarizes existing information about the role of purinergic signaling in the attainment and preservation of cancer cell phenotype. Purinergic signaling in the TME exerts autocrine–paracrine actions to alter energy metabolism to facilitate cell survival, proliferation, migration, immunosuppression, and drug resistance in cancer cells. exATP is an emerging inducer and regulator of drug resistance through EMT and CSCs in cancer cells, and thus, targeting exATP will improve drug sensitivity of cancer cells to chemotherapeutic agents (363). Clinical interventions of purinergic signaling agonists and antagonists in various cancer patients are ongoing (**Table 1**), but are still in their infancy as very few agonists and antagonists are successful and steady *in vivo* (364). This may be due to the fact that most purinoceptors are ubiquitous and hence there is a problem of redundancy, and selectively targeting specific cell type remains a challenge.

Thus, there is still a lot unknown about purinergic signaling. For example, why do dual actions of exATP and purinoceptors occur in both normal and malignant cells? What is an apparent function of various purinoceptors and the concrete effects mediated by purinoceptors? What is the cell-specific location of ectonucleotidase expression in a tumor and is this expression stromal or in the immune cells? Furthermore, what is the most effective route of administration of ATP or purinoceptor-selective agonists or antagonists in humans, which best imitate the current administration procedure for anti-cancer drugs? Thus, further understanding of purinergic signaling and its interactions with other oncogenic signaling systems is the need of the hour.

## Author contributions

JK conceptualized the manuscript, reviewed the literature and wrote the manuscript. SD did the literature search. All authors contributed to the article and approved the submitted version.
